# Capillary electrophoresis applied to DNA: determining and harnessing sequence and structure to advance bioanalyses (2009–2014)

**DOI:** 10.1007/s00216-015-8703-5

**Published:** 2015-05-03

**Authors:** Brandon C. Durney, Cassandra L. Crihfield, Lisa A. Holland

**Affiliations:** C. Eugene Bennett Department of Chemistry, West Virginia University, Morgantown, WV 26506 USA

**Keywords:** DNA, DNA aptamer, Capillary gel electrophoresis, Sieving matrix

## Abstract

This review of capillary electrophoresis methods for DNA analyses covers critical advances from 2009 to 2014, referencing 184 citations. Separation mechanisms based on free-zone capillary electrophoresis, Ogston sieving, and reptation are described. Two prevalent gel matrices for gel-facilitated sieving, which are linear polyacrylamide and polydimethylacrylamide, are compared in terms of performance, cost, viscosity, and passivation of electroosmotic flow. The role of capillary electrophoresis in the discovery, design, and characterization of DNA aptamers for molecular recognition is discussed. Expanding and emerging techniques in the field are also highlighted.

## Introduction

**Fig. 1:**
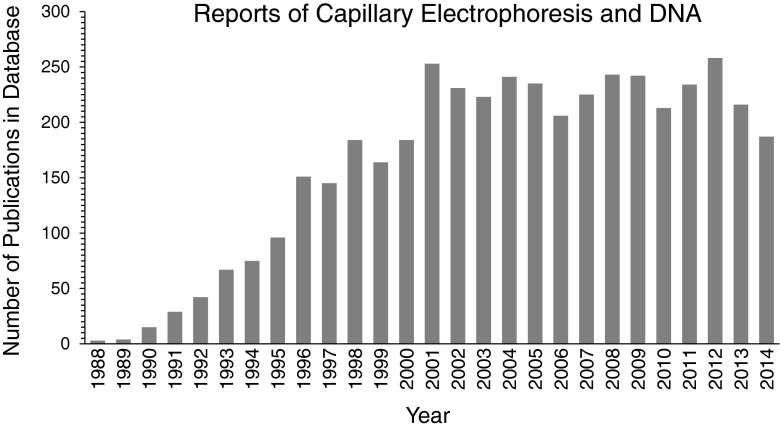
Number of publications on capillary electrophoresis separations of DNA in the SciFinder® database

Capillary electrophoresis separations are significant because they provide fast separations of limited sample volumes. Following reports of outstanding separation efficiencies of amines, amino acids, and peptides achieved using a 75-μm-inner-diameter glass capillary [1, 2], the technology was quickly adapted to DNA [3, 4]. The rapid growth and sustained use of capillary electrophoresis for DNA analyses is best illustrated by the number of annual journal publications, which is summarized in Fig. 1. Critical innovations reported early in the method development [5–10] dramatically increased the applicability to sequence and size DNA. Landmark applications include genome sequencing [11], forensic analysis of DNA with commercial systems [12, 13], and lab-on-a-chip [14–17]. In addition to sizing DNA, capillary electrophoresis has played a pivotal role in the generation of DNA aptamers and the quantification of aptamer binding affinity.

As the technology matures, research-driven advances have been transformed into heavily utilized applications, generating a large user-base focused on applying the methodology. Perhaps the best indicator of progress in the field of capillary electrophoresis DNA separations is the translation of this technology into the teaching laboratory [18, 19]. Capillary electrophoresis separations of DNA have been integrated in teaching exercises in genomic identification of food with a commercial chip [18]. Despite the maturity of this technology innovative research and new applications are reported. The goal of this review is to summarize developments in the use of capillary electrophoresis for DNA analyses. This paper includes capillary electrophoresis techniques reported from 2009 to 2014 that address critical barriers. The review begins with a brief discourse on the mechanisms relevant to DNA separations.

## Separation

Capillary electrophoresis is a high-throughput separation method commonly employed for DNA analysis owing to rapid analysis times and small sample volumes. Various modes of capillary electrophoresis, which are summarized in Table 1, are used depending upon the application. Free-zone and gel-facilitated sieving are the most commonly reported modes for DNA analyses. Unfortunately, free-zone capillary electrophoresis methods for DNA separations are limited because of the similar charge-to-size ratio of fragments of different length. To circumvent this problem gels are incorporated in capillary electrophoresis separations to sieve DNA fragments on the basis of size.

**Table 1:**
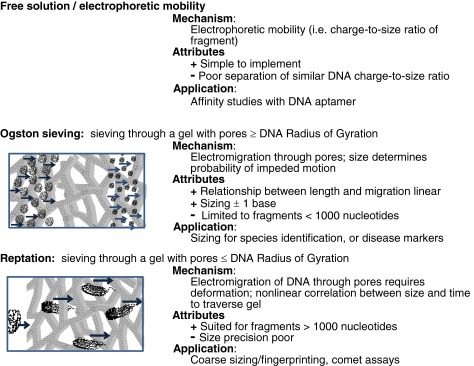
Capillary/microchip electrophoresis mechanisms

### Gel-facilitated sieving

Gel-facilitated sieving is one of the most commonly used methods for sequencing DNA in parallel analyses and sizing. Electroosmotic flow is suppressed by coating the surface of the capillary, and then the capillary is filled with sieving gel. Ogston sieving and reptation are the mechanisms of DNA transport through the gel. In Ogston sieving, shown in Table 1, DNA behaves as an incompressible sphere. The separation, which occurs with suppressed electroosmotic flow under reversed polarity, is based on the ability of DNA to pass unobstructed through the pores formed by the gel matrix. Smaller fragments of DNA migrate faster than longer fragments, and a linear relationship is observed between fragment size and migration time. Both sequencing and sizing are conducted within the Ogston regime. Reptation, which is depicted in Table 1, occurs when a DNA molecule is too large to pass freely through the pores of the gel and must deform or unfold to fit through the matrix. With this mode of sieving, the relationship between migration time and DNA fragment size is non-linear and peak resolution is worse, which makes sizing difficult. The fragment size at which the separation transitions from Ogston sieving to reptation can be approximated with a DNA size ladder and determined experimentally. A variety of matrices for DNA separations have been reviewed [20–26].

### Characteristics of DNA sieving gels

Prevalent matrices used for sequencing or sizing from 2009 to 2014 are summarized in Table 2. The factors that determine which separation matrix is utilized for a specific application are included in the table. The separation performance, which is the most critical figure of merit of a sieving gel, is measured by the chromatographic resolution and the upper size limit for Ogston sieving. Chromatographic peak resolution (Rs), is defined as Rs = (Δ*t*)/*W*_ave,_ where Δ*t* is the difference in migration times of two adjacent peaks and *W*_ave_ is the average width of the peak at the base (estimated as 4σ) [27, 28]. Resolution is more often calculated using the width at half-height [29, 30], which for a Gaussian peak is 2.35σ, because it obviates practical issues associated with a noisy baseline or overlapping peaks [29]. For Gaussian peaks this calculation generates the same value as obtained using the width of the base [27]. Peak resolution can also be described in terms of the minimum number of nucleotides that are distinguishable for two DNA fragments. This is calculated by dividing the difference in the number of bases for the two adjacent peaks by the calculated chromatographic resolution. For simplicity, the resolution that can be expected for different sieving matrices is reported in Table 2 in terms of the size in bases by which fragments can reliably be distinguished from one another.

**Table 2:**
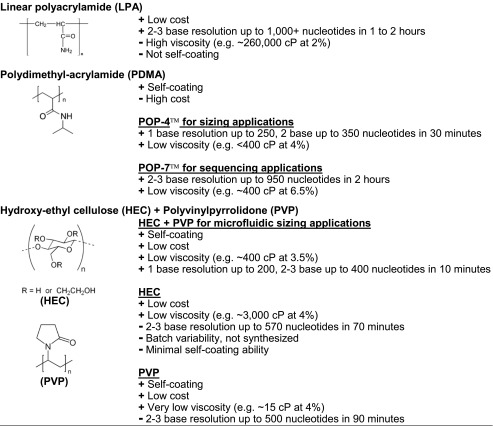
Gels used for DNA sequencing and sizing applications

In addition to separation performance, the cost, viscosity, and coating capability of a gel must also be considered. A gel that is easily synthesized or readily available at a low cost is preferred. The viscosity of the gel matrix is also critical because high pressure systems are required to introduce and remove viscous gels from capillaries. Such high pressures are incompatible with microfluidics. Coating the capillary or channel surface is necessary to suppress the electroosmotic flow. The coating must be stable and the effect on electroosmotic flow reproducible.

Linear polyacrylamide is used in capillary gel electrophoresis techniques owing to outstanding performance and low cost. The disadvantages of linear polyacrylamide are the high viscosity and inability to coat the surface of the capillary. Linear polyacrylamide is among the most viscous matrices used in DNA analysis; however, under a sheer force of 1.32 s^−1^, the viscosity of a 2 % linear polyacrylamide gel drops to 27,000 cP [31]. A suppressed electroosmotic flow is required, and different strategies for surface modification have been reported [32–34].

Linear polyacrylamide matrices were used for various applications between 2009 and 2014. The synthesis and optimization of two linear polyacrylamide matrices for the capillary electrophoresis separation of DNA fragments with less than 70 bases was reported and applied to size PCR markers for wild-type and mutant gastric cancer tissues with a resolution below five bases [35]. A 5 % linear polyacrylamide matrix was used in an integrated microfluidic lab-on-a-chip platform for DNA extraction, amplification, separation, and detection from a crude biological sample, and a full profile of short tandem repeats (STRs) was obtained for a standard DNA template in a 40-min analysis time [34]. Other microfluidic platforms utilizing linear polyacrylamide were employed for the analysis of *E. coli* [36–38], *Staphylococcus aureus* [37, 39], *Salmonella typhimurium* [37], human respiratory viruses [40], *Alu* insertions used for gender and ethnicity determination [41, 42], p53 gene mutations [43], and EndoV/DNA ligase mutations [44].

Polydimethylacrylamide matrices overcome the two major limitations of linear polyacrylamide sieving gels: viscosity and coating ability. The most prevalently used polydimethylacrylamide matrix is performance optimized polymer 4 (POP-4™), which contains 4 % polymer with 5 % 2-pyrrolidinone and 8 M urea [45]. Single-base resolution of DNA fragments up to 250 bases and two-base resolution up to 350 bases have been demonstrated within a 31-min separation for forensic DNA applications [46]. Other POP™ matrices containing higher percentage polymer concentrations have been optimized for sequencing applications. Matrices of 6.5 % polydimethylacrylamide have a viscosity between 75 and 1200 cP depending on whether the low or high molecular weight polymer is used in the synthesis reaction [47]. The POP-7™ formulation has a viscosity of only 395 cP [48]. The low viscosity is an advantage of polydimethylacrylamide. Unlike linear polyacrylamide, polydimethylacrylamide can coat the surface, so other coating materials or modifications are not required. The advantages of using a polydimethylacrylamide sieving matrix come at a cost, as it is the most expensive matrix available with POP-4™ (cat. # 402838 or # 4363752) available at a cost of approximately US$60 per mL [49]. It is also expensive to synthesize a polydimethylacrylamide matrix using the dimethylacrylamide monomer, but has been shown to yield comparable separation performance to commercially available matrices of polydimethylacrylamide in capillary electrophoresis [50] and linear polyacrylamide in a microfluidics platform [51]. The material is heavily used in forensics applications. New integrated microfluidics have been applied to methylated DNA using polydimethylacrylamide sieving gel to identify whether a forensic sample source was tissue [52], body fluid [53], or semen [54] and has been utilized to analyze seminal stains as old as 56 years [55], as well as analyzing polymorphisms of STRs [56].

Matrices composed of polydimethylacrylamide are also employed for a variety of applications outside of forensics. Sizing DNA with new matrices [57] and microfluidic platforms developed for STR analysis [58, 59] are often compared to bench-top analyses achieved using polydimethylacrylamide matrices. Applications of polydimethylacrylamide matrices outside of forensics include multi-locus variable number repeat analysis to genotype several bacteria including *Shigella* spp. [60], *Streptococcus agalactiae* [61], *Staphylococcus aureus* [62], *Clostridium difficile* [63]*, Listeria monocytogenes* [64], *Legionella pneumophila* [65], *Pseudomonas aeruginosa* [66], and *Francisella noatunensis* [67], and study the association of specific point mutations with susceptibility to particular antimicrobial agents [68]. These matrices also have merit in the agricultural field having aided in methods for sizing biomarkers for the identification of seven pathogenic species in bovine milk [69]. They have also been used in distinguishing genetically modified cotton and soybean [70], and single strand conformational analysis for the identification of seven infectious disease-causing pathogens [71].

Hydroxyethylcellulose, a polysaccharide-based gel derived from cellulose, is a low cost and low viscosity matrix; however, with this matrix the electroosmostic flow is not eliminated but is only suppressed by 20 % [72]. A drawback to utilizing hydroxyethylcellulose is polydispersity of the polymer chain because it is a naturally occurring polymer. Hydroxyethylcellulose matrices cost approximately US$0.14 per gram [73] with low viscosities at dilute concentrations. An early application of hydroxyethylcellulose for DNA separations yielded two-base resolution at an upper size limit of 570 bases using a 2 % matrix composed of polymer with a molecular weight range of 90–105 kDa that had been purified using an ion-exchange resin [74]. The viscosity of hydroxyethylcellulose matrices can be adjusted so that it can be suited for separating DNA of different size ranges by varying the percentage of low and high molecular weight hydroxyethylcellulose in the preparation [75]. A lower molecular weight, 90-kDa hydroxyethylcellulose matrix was used for the identification of genetically modified maize with DNA markers less than 200 bases [76]. A blended hydroxyethylcellulose matrix consisting of 0.21 % 27-kDa and 0.07 % 1-MDa hydroxyethylcellulose with 0.12 % 7-MDa linear polyacrylamide was used for the separation of DNA fragments ranging from 200 to 40,000 bases in 2 min in a glass microfluidic coated with polyhydroxyethylacrylamide [77]. The poor surface passivation by hydroxyethylcellulose can be overcome by blending other effective surface coating agents such as polyethylene glycol [78], polyvinyl alcohol [79], and polydimethylacrylamide [79].

Polyvinylpyrrolidone is a sieving matrix with mediocre separation performance, but excellent surface coating properties, low cost [80], and low viscosity, which can range from only 3 to 27 cP [81]. Polyvinylpyrrolidone matrices have been reported to demonstrate the feasibility of using short capillaries [82], performing portable methods [83], and improving detection through base stacking and field gradients [84]. Although this matrix is not widely used, a newly developed blended sieving matrix comprised of 20 % polyvinylpyrrolidone and 80 % hydroxyethylcellulose [57] harnesses the complementary properties of each material. Polyvinylpyrrolidone is an excellent coating material, and hydroxyethylcellulose provides better separation performance. The viscosity of the mixture is lower than a matrix containing only hydroxyethylcellulose and has been used for more than 90 consecutive capillary electrophoresis runs without deterioration in separation performance [85]. The matrix is mainly used in microfluidic platforms for DNA sizing for human identification STR analysis because it provides single-base resolution up to 200 bases and two- to three-base resolution up to 400 bases in a 15-min separation [58].

## Pivotal applications of capillary gel electrophoresis for DNA sieving

### Beyond de novo genome sequencing

Next-generation sequencing strategies are now commercialized as cheaper and faster alternatives based on highly multiplexed analysis of short reads [86]. However, Sanger sequencing via capillary gel electrophoresis is still commonly used to correct for errors in assembling the sequence data, for example in long repeats of DNA polymers. Thus, capillary gel electrophoresis is reported as an analytical technique used to assist in genome sequencing with next-generation sequencing technology. Capillary gel electrophoresis is used to improve quality control in next-generation sequencing [87], or to quantify the DNA library [88]. Droplet microfluidics was used in conjunction with capillary gel electrophoresis to ensure that a suitable amount of DNA is generated by PCR without a bias in size distribution [89].

#### End-labeled free-solution electrophoresis

End-labeled free-solution electrophoresis relies upon free zone capillary electrophoresis to separate DNA fragments that are covalently attached to a large molecule, such as synthetic peptoids [90] or proteins [91], often referred to as drag-tags. DNA mobility decreases with the fragment size, so smaller fragments migrate slower than larger fragments due to a decrease in the charge-to-friction ratio. Decrease in the polydispersity of proteins used for drag-tags decreases variation in charge and size distribution, which extends the sequencing read length [91]. Increase in the charge on the drag-tag increases wall interactions, which increases band broadening [92, 93]. Micelle drag-tags have been utilized in commercial capillary electrophoresis instruments and microfluidics to improve the readout time by optimizing the electroosmotic counterflow [94]. Additional applications for drag-tag methods include hybridization assays with short single-strand DNA targets [95] for the detection of single nucleotide polymorphisms (SNPs) [96] or to assess the formation of primer dimers in multiplex PCR reactions [97].

### DNA sizing

Beyond the use of capillary gel electrophoresis for DNA sequencing of the entire human genome, capillary gel electrophoresis continues to play a significant role in assigning STRs for human identification or detecting pathogen biomarkers. Repetitive sequences within the genome are harnessed to uniquely identify specific biomarkers for a number of applications relevant to pathogen detection, human disease, and especially human identification. DNA analyses are critical to forensic laboratories around the world. Human identification methods are based on the analysis of DNA sequences known as STRs, which contain two to five base repeats. For example, the STR Penta E has the recurring sequence AAAGA, and can vary among individuals from five to as many as 24 repeats of AAAGA. An individual will have two different sets of this repeat, one copy from each parent. Thirteen different STRs are used in the Federal Bureau of Investigation (FBI) combined DNA index system, also known as CODIS, for probability matching of a DNA sample to a specific individual. In a single forensic analysis a minimum of 16 markers are separated and sized. Four different fluorescent labels (e.g., FAM, JOE, TAMRA, ROX) are used to distinguish the STRs because some have similar length and overlap in separation space. Therefore, these overlapping lengths are resolved spectrally. The amplicons reflect the number of repeats at a specific locus, and the assignment of size is accomplished using a standard DNA ladder. These overlapping fragments are separated in the polydimethylacrylamide sieving gel matrix. The relationship between migration time and fragment size is linear. Sizing is used for genotyping through the identification of markers that have specific lengths and DNA sieving gels are critical to these separations.

#### Chip-based forensics

**Fig. 2:**
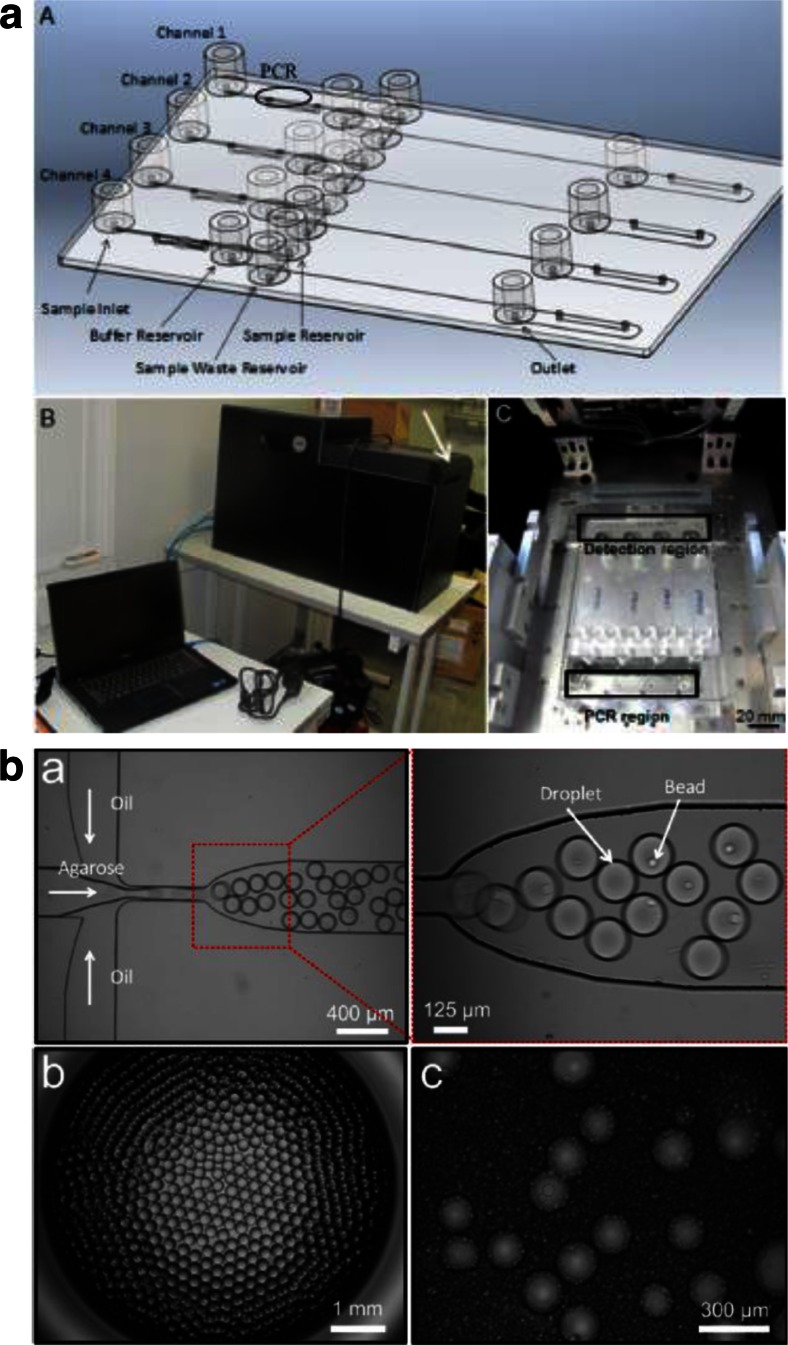
Examples of microchip devices for integrated forensic analysis of DNA. **a** An integrated microchip fabricated for online sample extraction, PCR amplification, and separation. Reprinted with permission from Le Roux D, Root BE, Reedy CR, Hickey JA, Scott ON, Bienvenue JM, Landers JP, Chassagne L, de Mazancourt P (2014) DNA analysis using an integrated microchip for multiplex PCR amplification and electrophoresis for reference samples. *Analytical Chemistry* 86(16):8192–8199. Copyright 2014 American Chemical Society. **b** The process of separating cells into oil droplets using a microfluidic droplet generator for single-cell processing and analysis is depicted. Reprinted with permission from Geng T, Novak R, Mathies RA (2014) Single-cell forensic short tandem repeat typing within microfluidic droplets. *Analytical Chemistry* 86(1):703–712. Copyright 2014 American Chemical Society

Microfluidic systems for forensic analyses continue to advance. Newer device designs generate results from buccal cells in under 3 h [98] or 4 h [58]. DNA from whole blood was processed using a device to integrate solid phase extraction with a 1.2 μL PCR chamber [99]. A simple disposable chip fabricated in 10 min using a printer and polyester toner at a cost of US$0.15 was reported [100]. A more sophisticated plastic microchip was reported for integrated sample extraction, PCR amplification, and DNA separation (Fig. 2A) and achieved single-base resolution of buccal samples with only a 7-cm separation channel [101]. The plastic device is cost-effective and was used over a 6-month period. In another report a microfluidic droplet generator, shown in Fig. 2B, was used for high-throughput isolation of single cells prior to integrated extraction, amplification, and sizing [102]. This approach of isolating single cells circumvents issues of analyzing and interpreting data obtained with DNA from multiple donors.

#### Self-assembled gels

**Fig. 3:**
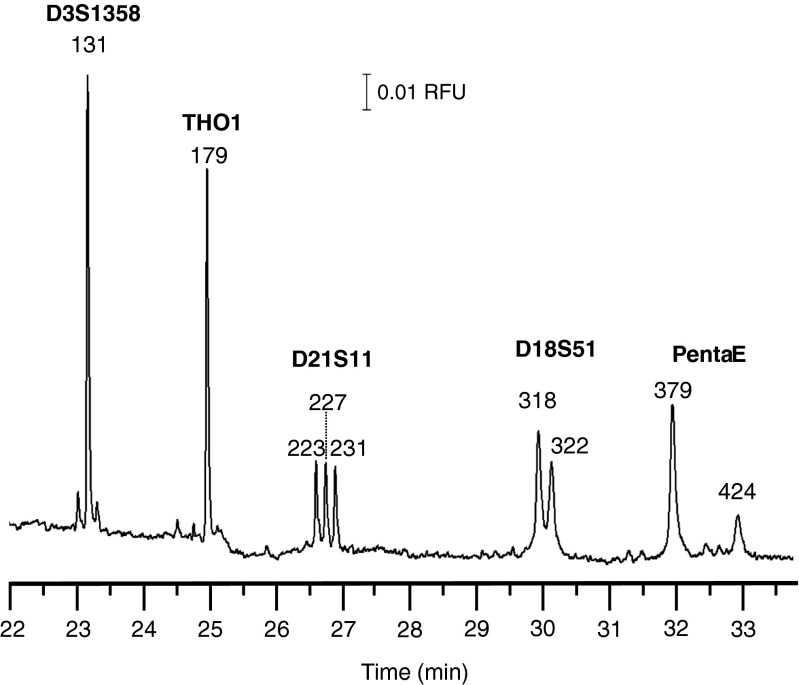
Separation of the 5 FAM labeled STRs produced from the multiplex PCR amplification of standard DNA template K562. Reprinted with permission from Durney BC, Lounsbury JA, Poe BL, Landers JP, Holland LA (2013) A thermally responsive phospholipid pseudogel: tunable DNA sieving with capillary electrophoresis. *Analytical Chemistry* 85(14):6617–6625. Copyright 2013 American Chemical Society.

Self-assembled gels with tunable selectivity are an alternative to POP-4™ gels. The phospholipids dimyristoyl-*sn*-glycero-3-phosphocholine (DMPC) and 1,2-dihexanoyl-*sn*-glycero-3-phosphocholine (DHPC) spontaneously forms a thermally reversible nanogel for DNA sizing [103, 104]. The nanogel adopts a bilayer nanodisk morphology at 19 °C which corresponds to a low loading viscosity of 50 cP for a 20 % solution [105]. When the temperature is increased to 30 °C the phospholipids assume a nanoribbon-like structure that forms a higher viscosity interconnected network. The DMPC-DHPC preparation is self-coating, suppressing electroosmotic flow by simply flushing the capillary with the phospholipid [106]. The phospholipid nanogel is roughly one-third of the cost of POP-4™ gels used for human STR analyses [107–109]. The nanogel separation shown in Fig. 3 is of FAM-labeled STRs from the PowerPlex® 16 analysis kit [103]. Single-base resolution up to 250 bases was demonstrated with a 10 % phospholipid nanogel [103]. Nanogels diluted to 2.5 % extend the range for precise DNA sizing up to 1500 base pairs [110]. The thermally responsive viscosity can support sieving gradients [103] as well as stacking cartridges that preconcentrate DNA upon injection and can be thermally erased prior to separation [110].

## Capillary electrophoresis and DNA aptamers

### Generating DNA aptamers

**Fig. 4:**
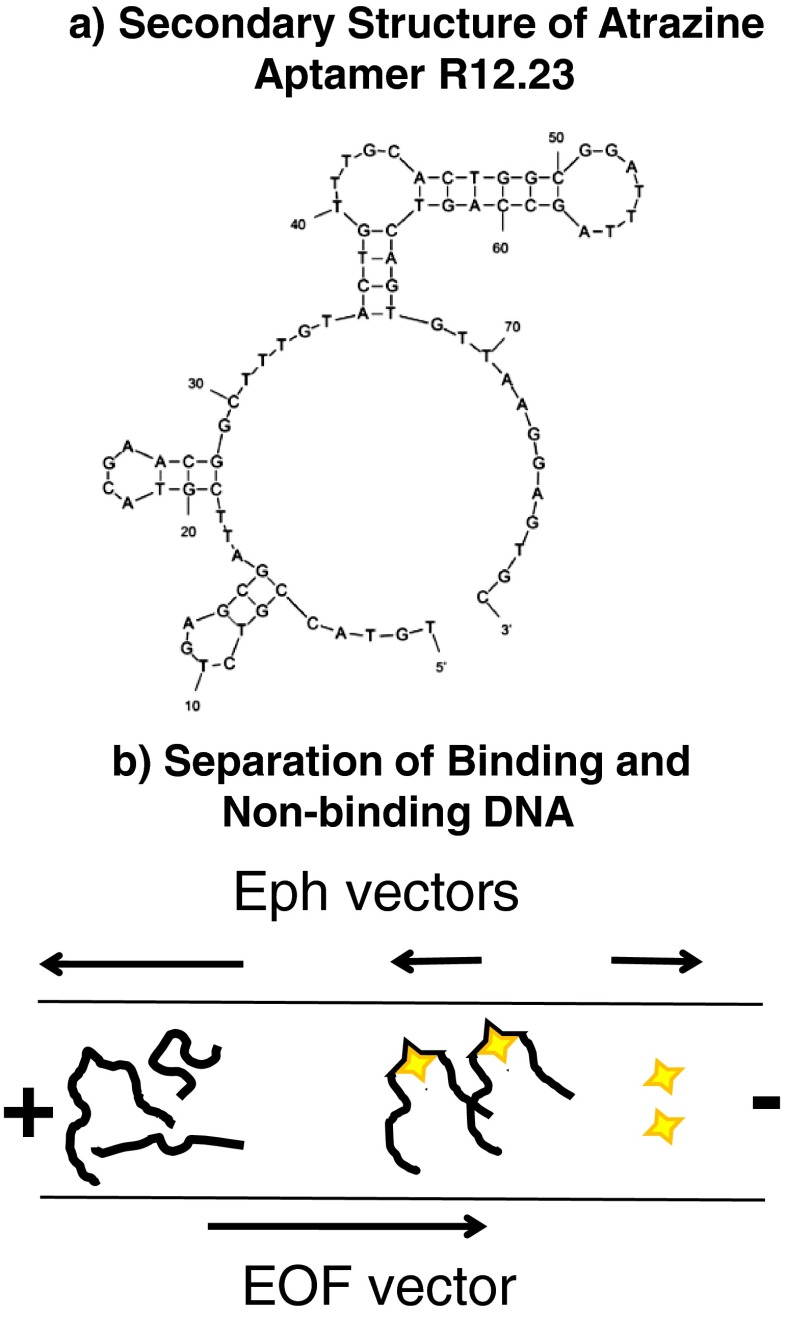
**a** An atrazine aptamer is used as an example of how DNA aptamers exploit secondary structure of single-stranded DNA to bind a target. Reprinted from Williams RM, Crihfield CL, Gattu S, Holland LA, Sooter LJ. In vitro selection of a single-stranded DNA molecular recognition element against atrazine (2014) *International Journal of Molecular Sciences* 15(8):14332–14347, available under a Creative Commons Attribution License. **b** The change in migration resulting from aptamer binding to target is depicted

DNA recognition elements, also called aptamers, can be tailored to bind biomolecule targets with selectivity and specificity approaching that of antibodies. An aptamer of single-stranded DNA spontaneously forms secondary structure that leads to strong aptamer–target molecular binding. Figure 4A depicts the secondary structure of an atrazine aptamer [111] as predicted by *m*-fold [112]. Unlike antibodies, aptamers are stable under the conditions required for robust biosensors. Once aptamer–target binding is realized the temperature can be manipulated to release the target and then refold the aptamer. This thermal reversibility of aptamer structure can be harnessed to reset the biosensor and use it repeatedly. DNA aptamers are produced through a process of iterative enrichment of the DNA–target complex from a DNA library through a method called systematic evolution of ligands by exponential enrichment (SELEX) [113]. Higher-throughput separation techniques play a significant role in the enrichment process. The unique separation capabilities of capillary electrophoresis have led to new strategies for aptamer generation and provide a quantitative means to measure binding affinity of DNA aptamers.

**Fig. 5:**
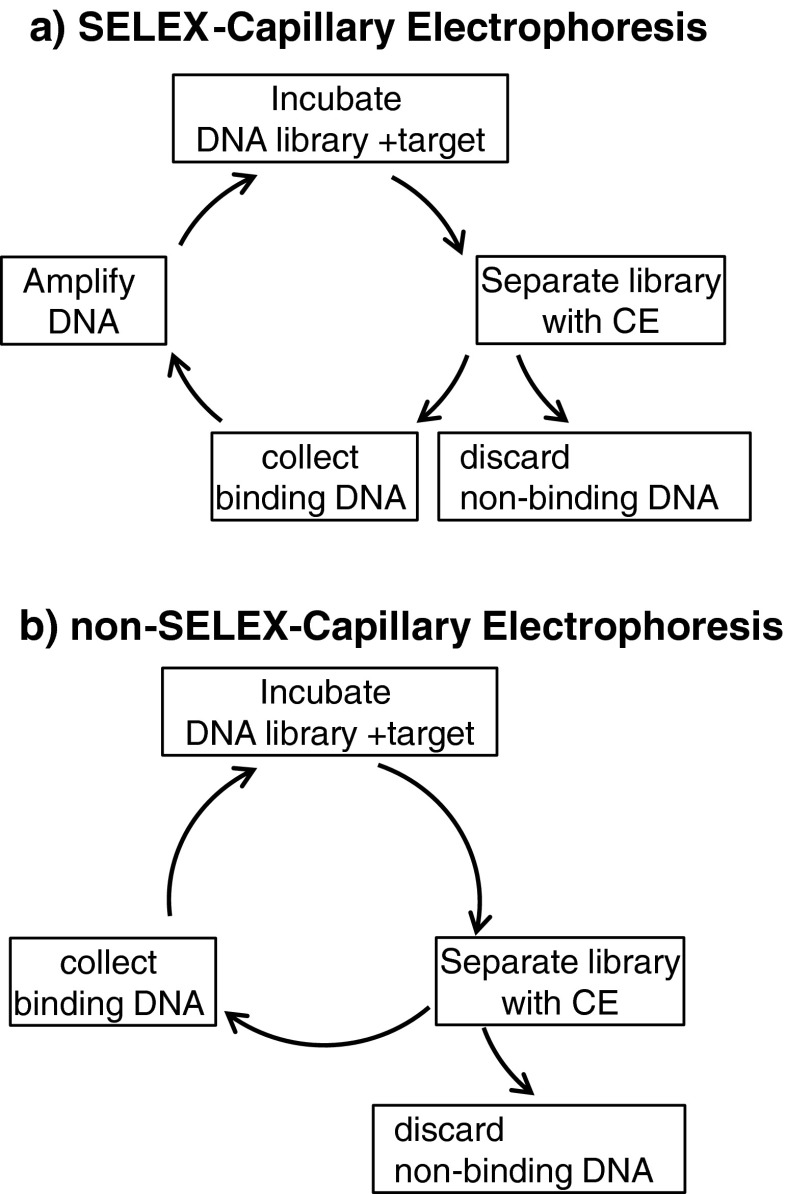
**a** Aptamer discovery via capillary electrophoresis-SELEX requires cycles of incubation of DNA with target, removal of non-binding DNA, and amplification of binding DNA. **b** Aptamer discovery via non-SELEX-capillary electrophoresis reduces the time required by removing the amplification step between each incubation with target

**Fig. 6:**
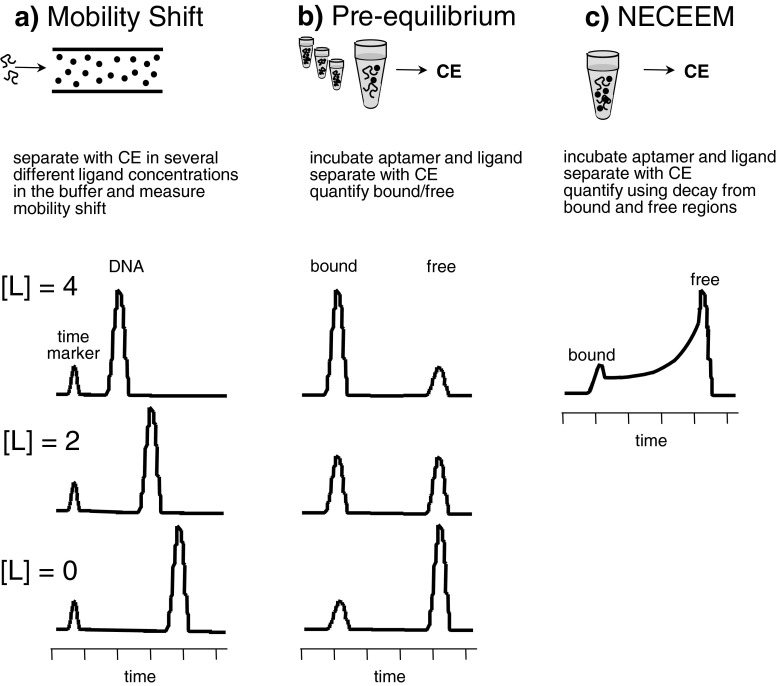
Obtaining a binding constant for aptamer using **a** mobility shift, **b** pre-equilibrium, or **c** nonequilibrium capillary electrophoresis methods. **a** Aptamer and ligand interaction occurs in-capillary, and the binding constant is determined on the basis of the shift in migration time correlating to the ligand concentration. **b** Aptamer and ligand interaction occur off-capillary, and the amount of bound and free aptamers is monitored in relation to ligand concentration. **c** In nonequilibrium capillary electrophoresis of equilibrium mixtures, aptamer and target are in equilibrium off-capillary. Dissociation begins during separation. Binding constants are determined using the decay from the bound and free regions of the electropherogram

Capillary electrophoresis is used to generate aptamers for targets with an electrophoretic mobility different from that of DNA fragments. The similar electrophoretic mobility of DNA fragments in free zone capillary electrophoresis separations is considered a disadvantage for DNA separations, but is harnessed for this technique. Upon binding to the target, the DNA–target complex undergoes a change in electrophoretic mobility, which shifts the migration time of the complex from that of the non-binding DNA fragments. Figure 5B depicts this change in migration when the target is either positively charged or neutral and the experiment does not suppress electroosmotic flow. The integration of capillary electrophoresis in the SELEX process, as first described by Mendonsa and Bowser [114], is illustrated in Fig. 6A. Non-binding fragments co-migrate in a single unresolved band, whereas binding fragments migrate before or after the non-binding band depending on whether the target molecule increases or decreases the mobility of the DNA–target complex. Fractions are collected from the capillary so that they may be amplified and further enhanced by repetitive rounds of positive or negative selection. Advantages of capillary electrophoresis-SELEX over other SELEX methods are a smaller sample handling volume, faster screening, and most importantly no need to immobilize either the aptamer or the target during selection rounds.

### Capillary electrophoresis-SELEX

Capillary electrophoresis-SELEX methods [115–120] have recently been used to generate aptamers predominantly for proteins, although work with peptides [121] and small molecules [122] is reported. Innovations in the field of capillary electrophoresis-SELEX continue. The method has been translated into a micro free-flow electrophoresis, which is a two-dimensional device in which electrophoresis is applied perpendicularly to hydrodynamic flow to separate binding and non-binding DNA fragments [116]. Integrating the selection into a microfluidic device further reduces the volume required for fraction collection with next-generation sequencing. Previously, observations of unexpected decreases in the rate of aptamer enrichment with successive rounds were attributed to the appearance of short DNA by-products [123]. Bias in the PCR amplification of non-binding fragments over bound fragments leads to inaccurate selection of aptamer candidates. Drawbacks of PCR amplification have led to the use of real-time PCR to decrease the number of enrichment cycles [118].

The method of capillary electrophoresis-SELEX has been adapted to reduce the repetitive selections required to enhance binding so that only a single amplification step is utilized after the capillary electrophoresis enrichment. This alternative method of capillary electrophoresis–non-SELEX integrates DNA selection without amplification as outlined in Fig. 6B [124]. With non-SELEX capillary electrophoresis, regions of free and bound DNA are not fully resolved. The dissociation is evaluated by assessing the relative amount of DNA that is bound, free, or dissociates during the capillary electrophoresis separation [125]. Predictive models have been developed to optimize the approach [126]. Non-SELEX methods have recently been used to generate aptamers for proteins [127–137], although work with lipopolysaccharide [138] is also reported. Emulsion PCR has been reported to reduce accumulation of DNA by-products [132]. The method continues to be refined by combining in-capillary mixing of reactants and affinity analysis [127], and passivating the capillary surface [137]. Other adaptations of capillary electrophoresis for aptamer generation include the integration of reproducible fraction collection with on-column non-covalent fluorescent labeling [139] and next-generation sequencing [140] with capillary isotachophoresis. Innovations in automated fraction collection using ink jet printers and 96-well microtiter plates will further improve the method [119].

### Evaluating DNA aptamer binding

Capillary electrophoresis is a powerful tool to quantitatively measure dissociation constants for aptamers with targets, and excellent reviews of affinity binding methods for aptamers and targets are available [141, 142]. Capillary electrophoresis is well suited to evaluate aptamer affinity when the electrophoretic mobility of free and bound aptamer differ significantly because the separations are fast and require working sample volumes of 15 μL to deliver injection sample volumes of 2 nL or less. Methods of assessing dissociation constants with capillary electrophoresis are based on a change in the charge-to-size ratio upon binding, which leads to mobility shift that depends on the rate of association. Protein–aptamer complexes have a large change in mobility, which can be easily detected with electrophoresis. The most common methods are classical mobility shift affinity capillary electrophoresis [143], pre-equilibrium affinity capillary electrophoresis [144], or nonequilibrium capillary electrophoresis of equilibrium mixtures [145]. These processes are depicted in Fig. 6 and discussed in greater detail below.

#### Classical mobility shift affinity

Classical mobility shift affinity, shown in Fig. 6A, involves the separation of an aptamer in a background electrolyte devoid of target and then in background electrolyte with increasing concentrations of target. The concentration of aptamer and target determines the amount of time the aptamer is complexed as it migrates in the capillary. The migration time will shift with increasing target concentration as a function of the dissociation constant. An advantage to using measurements of mobility shift to measure aptamer dissociation constants is that the experiment can be used when it is difficult to estimate the concentration of the target (e.g., bacteria [146]). Mobility shift affinity capillary electrophoresis is best suited to aptamer complexes with weak or intermediate binding affinity in order to sample a range of complex migration shifts resulting from fractional complex formation within the time frame of the separation. As a result, the use of mobility shift affinity capillary electrophoresis is not reported frequently to measure aptamer–target affinity, although it has been used to evaluate the effect of interactions between aptamers and metal ions [147].

#### Pre-incubation equilibrium affinity capillary electrophoresis

Pre-incubation equilibrium affinity capillary electrophoresis (Fig. 6B) requires that aptamer and target are incubated off-capillary. Different concentrations of the target are incubated in a constant concentration of the aptamer. Each incubation shown in Fig. 6B is separated by capillary electrophoresis to quantify the bound and free concentration of aptamer. Pre-equilibrium affinity capillary electrophoresis is used when the aptamer complex does not dissociate significantly during the time frame of the separation. Pre-incubation equilibrium affinity capillary electrophoresis has been applied to proteins [114, 116, 117, 123, 148], peptides [121], and small molecules [117, 149]. An innovative application utilizes a micro free-flow device for affinity capillary electrophoresis as a means to sample the ratio of bound and free aptamer at a wide range of concentrations. The method uses concentration change due to lateral diffusion, internal standards, and two-dimensional detection to record concentrations from different line scans obtained throughout the separation [150].

#### Measurements based on nonequilibrium capillary electrophoresis

Measurements based on nonequilibrium capillary electrophoresis of equilibrium mixtures (Fig. 6C) involve injecting and then separating a mixture of target and aptamer in the capillary. The complex dissociates throughout the run and the resulting electropherogram does not contain discrete peaks that are baseline resolved. Instead the electropherogram contains zones of fully bound and free aptamer that define an intermediate region reflecting dissociation with increasing run time. Deconvolution of the unresolved peaks and mathematical manipulation provide both the binding constant and decay constant of the DNA aptamer–target [145] and can be utilized when the concentration of the target is unknown [151]. Nonequilibrium capillary electrophoresis of equilibrium mixtures provides information about the dissociation constant and the rate constants in a single run. The method is predominantly used to evaluate protein binding aptamers [115, 120, 124, 127, 129–133, 135–137, 145, 151–153], although measurements of peptides [134], small molecules including lipopolysaccharide [138], and quinine [154] have recently been reported.

## Future directions of emerging and expanding technology

Innovations in the development of capillary electrophoresis methods for DNA separations have emerged beyond sizing DNA, generating aptamers, or characterizing the affinity binding. Several recent research advances in mechanisms of DNA separations, novel sieving gels, and even artificial gels enable new areas of scientific discovery. A few of these techniques are highlighted as areas to watch for future expansion.

### Improved analyses based on composition

A recent report outlines a separation of the set of single-stranded DNA, 76 nucleotides in length differing in sequence by 2–5 bases per DNA strand. The separations were performed in running buffer containing different phosphate-derived sodium salts. The presence of guanosine 5'-monophosphate, adenosine 5'-monophosphate, uridine 5'-monophosphate, deoxyguanosine monophosphate, or phosphate supported sequence-based selectivity of DNA fragments which was suggested as an alternative to stability and conformation-based analyses [155]. Metal cation mediated-capillary electrophoresis, which is sensitive to conformational change [156], generates separation-based aptamer assays of 5 μM cocaine detection through conformational change associated with displacement of the aptamer target [157]. DNA aptamers can also be used as labels for indirect molecular detection. The concept of conformational change associated with aptamer–target displacement is utilized for multiplexed separation-based assays [158]. In the absence of the target, aptamers are cleaved by phosphodiesterase I [158]. Aptamer binding stabilizes the DNA aptamer and protects it from enzymatic cleavage [158]. Thus, intact fluorescently labeled aptamer strands indicate binding. The separation is multiplexed by using 23-, 36-, and 49-nucleotide-long aptamers to detect adenosine, ochratoxin A, and tyrosinamide [158].

### Improved analyses with transformable gels

Thermally responsive matrices, such as phospholipid nanogels [103, 110], are ideal for DNA analysis because the material can be loaded into a separation channel under conditions of low viscosity and then switched to a higher viscosity to accommodate the sieving separation. One class of separation matrix includes triblock copolymers of poly(ethylene glycol) (PEG) and poly(propylene glycol) (PPO) that have compositional formulas of PEG_*a*_PPO_*b*_PEG_*a*_ and are commonly known as Pluronics. The use of these materials as DNA separation matrices stems from an aqueous micelle structure that allows for higher concentration polymer solutions to be implemented for sieving while keeping a very low viscosity, e.g., a 15 % Pluronic F108 matrix has a viscosity of only 21 cP [159]. From 2009 to 2014 Pluronic matrices were predominantly used for single-strand conformation polymorphism analysis, when multiple fragments with the same length and only slight differences in sequence can be resolved for a cost as low as US$0.10 per gram [160]. These methods require a matrix that is non-denaturing allowing for the exploitation of subtle differences in mobility due to changes in secondary structure created by DNA sequence variability. The hydrophilic micelle structure of the polyethylene oxide chains in Pluronic matrices provides dynamic surface coating and favorable interactions with DNA analytes in solution, making it possible to resolve fragments on the basis of secondary structural differences. There are 48 total Pluronic formulations; however, only a few are transparent and can be used in conjunction with DNA detection methods [161]. The Pluronic F108 matrix was utilized for the detection of pathogens [159, 161–168] or human biomarkers [169, 170].

### Artificial matrices

Artificial matrices can be created with micrometer to sub-micrometer features fabricated within separation channels with electrically insulating materials. Channels with nanoscale dimensions, pillar arrays, and self-assembling colloidal crystals are examples of artificial matrices. Many studies utilizing pillar arrays examined the effects of geometry [171], size [172], order [173], and space [174, 175] on separation performance. Square and rectangular pillars with different orientations were used to show that electric field distribution, velocity, and motion are impacted by pillar geometry and packing [171]. Improvements in resolution have been obtained by decreasing pillar diameter [172], decreasing convective steering by increasing spacing [174], maintaining order of the array [173], and creating a more uniform electric field through the use of a nanofence rather than a traditional hexagonal pillar array [175]. Motion within sparse hexagonal ordered arrays has been proven to be driven by a non-uniform electric field [176], which causes conformational changes in DNA leading to band broadening [175]. Changing the angle of the applied electric field with post arrays expanded the range of applied voltages for separations and decreased the required separation length [177].

Studies utilizing fabricated obstacles provide an experimental means to elucidate basic principles of DNA separation and provide insight regarding transport. When the separation channel dimension is equal to the persistence length of the DNA molecule (45 nm) the mobility decreases with increase in DNA length [178]. The relationship holds true for channel dimensions 10 times greater than the persistence length (450 nm), and supports the assumptions of Ogston transport. However, the opposite trend for DNA mobility (i.e., increase in mobility with increase in DNA length) was observed for a channel of intermediate size (250 nm) [178]. Understanding this shift in mobility and how it relates to changes in entropy and the degree of interaction between the DNA and the channel wall provide a means to tune the size of a fabricated matrix around particular DNA applications involving separating a specific range of fragment sizes. Along with the physical barriers that impact DNA mobility, ionic strength of the buffer ties to the degree of electrostatic interactions and hydrodynamic confinement between the DNA and the wall of a nano-channel [179, 180]. The field-dependent mobility and DNA trapping mechanisms can be observed at high and low DC electric fields through the use of polyvinylpyrrolidone to decrease the overall width of a channel by forming rigid chain obstructions [181, 182]. Similar results were obtained using glass capillaries with a 750-nm inner diameter, obviating the need for high resolution lithography used to fabricate a nano-slit device [183]. In other studies, colloidal crystal suspensions have been utilized to create artificial matrices, which are more easily fabricated than pillar arrays or nano-slits. The use of monodisperse colloidal crystals is critical for creating uniform pore sizes for optimal resolution and reduced band broadening in comparison to a matrix composed of colloidal particles of differing size [184]. These technologies hold the potential to generate low-cost, high-performance, fabricated microfluidics for DNA analyses and eliminate the need for a gel sieving matrix.

Capillary electrophoresis plays a critical role in the development of DNA analysis technologies. It has been the method of choice for DNA analysis techniques commonly used for sequencing, sizing, and aptamer discovery and affinity studies. As novel approaches emerge, capillary electrophoresis techniques evolve from development stages to validated and applied methods. Still, the development of techniques to better understand separations in capillary, such as artificial matrices, suggests that optimization of capillary electrophoresis methods will continue to be pivotal in expanding the field of DNA analysis.
